# Impact of Covid‐19 pandemic on the transnationalization of LGBT* activism in Japan and beyond

**DOI:** 10.1111/glob.12423

**Published:** 2022-12-02

**Authors:** Sakura Yamamura

**Affiliations:** ^1^ Max Planck Institute for the Study of Religious and Ethnic Diversity Department of Geography RWTH Aachen University Aachen Germany

**Keywords:** diversity, Japan, LGBT*, social activism, transnational networks, transnationalization

## Abstract

The global Covid‐19 pandemic has strongly impacted social practices, relocating communications and social networks into the digital space. Contextualized in such impact of the Covid‐19 pandemic, the local LGBT* activism in Japan achieved a special momentum: both the acceleration of the socio‐spatial relocation of LGBT* activism to the digital space and the postponement of the Tokyo Olympics 2020 by 1 year enabled activists to mobilize people domestically and globally. The pandemic was not the actual cause or driver of the local LGBT* activism, yet it has been an important catalyst for the transnationalization of the local movement in Japan, pushing evidently the spatial boundaries to achieve broader public outreach but in turn also receiving stronger support from the global community through transnational networks. This study explores novel dynamics of spatiality and temporality of social transformations through the Covid‐19‐induced increase in global digital connectedness as well as transnationalization of local actions.

## INTRODUCTION

Amongst the different severe impacts which the Covid‐19 pandemic had on society, one of the most evident changes in people's daily lives has been the radically accelerated relocation of social interactions and exchanges onto virtual platforms. This change in socio‐spatial patterns from in‐person meetings in physical spaces to digitalized online spaces, questioning the relations of privacy and exclusivity of offline to online communications, has indeed impacted many globally (Lomanowska & Guitton, 2016; Nabity‐Grover et al., 2020). After 1 year of working daily from home and communicating through Zoom, Teams and other digital platforms, the digital space has become a substantial part of daily life for many of us. Though the overload of screen time has taken a toll on the physical and mental health of those working from home (Pfefferbaum & North, 2020; Stuart et al., 2021), the digitalization of events in this pandemic has also brought about the increased accessibility of events—be it professional or private‐social—and global connectedness beyond national borders. Now that online‐based communications have become ubiquitous, if not essential, to keep contact while physically distancing, social life has shifted strongly into the virtual world, and with it the usage of digital ethnography in research (Horst & Miller, 2012; Pink et al., 2015; Sade‐Beck, 2004). Not the pandemic itself, but the digitalization of everyday lives, that has been tremendously and forcibly accelerated by the pandemic, appears to have brought also substantial changes in the transnationalization of global society.

One such evidence of change and very likely sustainable transformation of social practices can be observed in emergent LGBT* activism in Japan, which is currently on the verge of experiencing a crucial turn coinciding with the Tokyo Olympics 2020 —postponed also due to Covid‐19 until summer 2021. With the diversification of societies in times of global migration, sexual orientations and gender identities of individuals bring another dimension of diversity to what has been discussed as superdiversity and the superdiversification of society (Vertovec, 2007; Yamamura, 2022). Though LGBT* movements might appear to be a local issue concerning individuals, LGBT* activists have long been supporting each other and seeking networks beyond national borders (see e.g. ILGA World, the International Lesbian, Gay, Bisexual, Trans and Intersex Association established in 1978), not least through increasing transnational migration (Ayoub & Bauman, 2019; Della Porta & Tarrow, 2004). Similar transnational activism connecting the local and the global has been discussed in recent research, too, yet the transnational connections tend to be limited to the same ethnic or national group (Koinova & Karabegovič, 2017; Li & Fung, 2021; Mercea, 2017). Such an approach does not reflect ongoing debates on the diversification of transnational communities themselves and the critique on the ethno‐focal lens (Meissner & Vertovec, 2015; Yamamura & Lassalle, 2020). Apart from this pitfall in transnationalism studies, what is missing in the discourses on transnational LGBT* activism is the focus on the actual dynamics between the local and such global movements. The transnational perspective on LGBT* activism should go beyond the dichotomy of the global and the local as separate entities and focus on the interwoven and interconnected transnational context instead.

LGBT* activism in Japan has found only limited attention within debates on queer activism so far. Recent studies on queer and queering Asia discuss the novel paradigm of the independent emergence of queer approaches as distinct from feminist studies (Chiang & Wong, 2017). Moreover, scholars have stressed the need for a shift from a Western view on LGBT* issues to a more Asian contextual view, therefore calling for ‘queer Asia’ as a method (Chiang & Wong, 2017; Yue, 2017). At the same time, such an understanding of Asian culture as holistic has also raised questions whether contexts such as Japan could be compared to those in South Korea or China (Eguchi, 2021). In fact, scholars working on Japanese queerness have pointed out the difficulties with such cross‐cultural comparisons (McLelland, 2005). While LGBT* issues in Japan had been described as developing independent from the Western context (McLelland, 2005), there has been a paradigm shift in 2015, which has been labelled an ‘LGBT boom’ (Horie, 2015). It signifies the discourse of Japanese LGBT* issues shifting from local to Anglicized terms and adopting Western‐based concepts (Fotache, 2019). This alludes to a certain transnationalization of the LGBT discourse in Japan. However, the activism has not been discussed from such perspective so far.

The extent of the transnationalization of LGBT* activism in Japan, however, has become especially more visible and accessible to people beyond its national borders given the radical relocation of LGBT* activism from the local to the virtual space during the pandemic. Against the backdrop of this novel shift, this study critically reflects on the impact of Covid‐19 on the transnationalization of such local activism. It embeds the change of LGBT* activism in times of the pandemic into the discourse on global networks from a superdiversity perspective. I analyse LGBT* activism in Japan, which is not only facing the Covid‐19 pandemic as any other country but also the (planned^1^) hosting of an international mega‐event, the Tokyo Olympics. While the Olympics are crucial in the overall narrative, being used as a momentum by LGBT* activists, it should merely be regarded an example of the paradigm shift towards stronger global connection and transnationalization from a superdiversity perspective. The study points to novel spatial and temporal social transformations brought about by the Covid‐19‐induced increase in global digital connectedness and by the transnationalization of actions to change local policies. It aims to contribute to the discourse on novel takes on global networks and transnationalization in the pandemic context from the perspective of transnationalism and superdiversity studies.

## THE BACKGROUND STORY: GENDER ISSUES AND LGBT* MOVEMENTS IN JAPAN

Japan is the only G7 country not to have legalized same‐sex unions so far. Also, gender equality indicators remain substantially below average compared to other Organization for Economic Cooperation and Development (OECD) member states (OECD, 2021). Briefly before the summer when the Olympics were to be hosted, the ex‐Prime Minister and Tokyo Olympic Committee (TOC) chair Mori resigned from his post over misogynist comments. Furthermore, mainly conservative politicians of the Liberal Democratic Party (LDP) also made headlines with discriminatory statements towards sexual and gender minorities. However, apart from these political scandals and dominant discourses on the lawmakers’ side, the population has become significantly more supportive of gender diversity. According to the most recent poll by one of the major newspapers (Asahi Shimbun Digital, 2021), 65% of the Japanese speak for the recognition of same‐sex marriage and 32% against the recognition of same‐sex marriage. The younger generation is especially very supportive, with numbers ranging up to 80% with the group of 19‐ to 29‐year‐olds, and even with 66% of those in the 60s still backing; the reaction of the elderly above 70, however, turns to strong disagreement. These polls do not necessarily directly reflect an increase in the support for gender and sexual diversity in the wider society during and through the pandemic. However, when considering the differences in the age groups, it can be assumed that there is indeed a tendency to a societal change towards supporting diversity in a long‐term perspective.

Despite the national government opposing this popular voice and not initiating the legalization of same‐sex‐marriage, since 2015 an increasing number of municipalities throughout Japan have begun issuing certificates of same‐sex partnerships. Although the certificates are legally not binding and also diverse in the actual schemes, these municipalities have administratively recognized same‐sex partnerships. The administrative recognition encourages further institutions and actors within the municipalities, such as hospitals or landlords, to treat such same‐sex couples as equal to married heterosexual couples. As of October 2022, 239 municipalities have introduced same‐sex partnership schemes, and 3456 couples have already registered (Nijiiro Diversity, 2021). While these partnership schemes are only valid only on the local level, and municipalities only recently started making agreements for mutual recognitions, three prefectures, that is, Ibaraki, Osaka and Gunma, have introduced prefecture‐wide partnership schemes valid even at the regional level. Two more prefectures are considering doing so in the near future. Another novel development is the introduction of so‐called partnership family systems, as introduced in three cities, where children are recognized as mutual children in a same‐sex partnership, thus allowing medical decisions and retrieving them from schools and kindergarten by the nonbiological parent.

Beyond these administrative novelties and an increasing trend towards opening up to gender diversity, there are also several court cases pending on same‐sex marriages and also transgender issues. Plaintiffs have commenced actions as early as 2018 and recently reached a historical ruling by the Sapporo^2^ district court in March 2021 that the nonrecognition of same‐sex marriage is unconstitutional. Although it was only a ruling from a district court, and further appeals are to follow to the Higher Court and eventually to the Supreme court, the LGBT* rights movement in Japan is winning an important momentum. Several more rulings are also expected in the near future from further district courts. Although these legal issues relate to national and local regulations, as the following case will show, local LGBT* activism is intertwined with global LGBT* supporters and actors, where resources from the transnational community are actively and strategically used. LGBT* activism has become inherently transnationalized. It reflects a growing migration‐led diversification of societies even in Japanese society, which is otherwise often perceived as being homogenous.

## COVID‐19 PANDEMIC AS A GAME‐CHANGER: RELOCATING TO A VIRTUAL WORLD AND WINNING MORE TIME

Both a blessing and a curse, the Covid‐19 pandemic has led to the increase in social interactions through digital devices in virtual spaces. Following social and political issues through social media before the pandemic, I had started noting an increasing presence of LGBT* and diversity‐related events on Facebook when deciding to more systematically conduct digital ethnography over social media (Horst & Miller, 2012; Pink et al., 2015; Sade‐Beck, 2004). Primarily following pages and organizations on Facebook, but also participating in a multitude of meetings on Zoom and other online platforms on issues of diversity at large (not only LGBT* but also migrant initiatives and transnational startup events), I was struck by how transnational the lived realities of LGBT* communities had become in Japan. Events in which I participated included, but was not limited to, those addressed at the LGBT* community itself, that is, online social gatherings and support, such as queer movie viewings with discussions or general peer discussions on the diversity agenda in sports. Events were also offered to a broader queer and allies audience with explanatory characters, for example, on legal cases with legal experts and scholars. Moreover, events were addressed at a more general audience aimed at fostering awareness for gender diversity issues, such as introductions to terminologies on sexual orientation and gender identities, or on gender equality issues within corporate social responsibility agendas. In contrast to events before the pandemic, these events were characterized by bilingualism (many being consecutively interpreted by community members) and the diversity of the origin of the participants, which have been repeatedly pointed out at the events and at follow‐up interviews. Along with tracking debates over more than a year of the coronavirus pandemic on Twitter and YouTube channels for specific events, such as the Tokyo Rainbow Pride, I closely followed and actively engaged at events of LGBT* and diversity‐related interest groups, ranging from formalized associations to informal online social groups. Indeed, such events used to be hosted in person before the pandemic, and the organizers of these events were often and explicitly thematizing the novel environment, apologizing for the technical glitches that would still occur. Furthermore, internal insights of the communications and participatory observations on the trainings for volunteers for the Olympic Games were achieved as I had been registered as a potential volunteer for the Paralympics. Analyses of policy documents, news media and official statements round up this empirical case.

What could be observed in times of Covid‐19 was a change in the public outreach of most kinds of local social groupings, including local LGBT* activism and diversity‐related events. These events would have originally been unreachable to audiences and peer groups outside of Japan, or even outside of the Tokyo metropolitan area. The online events reportedly reached participants beyond Tokyo, but also a far larger proportion accessed them from abroad (withstanding time difference). With only occasional announcements of local physical events on social media, activities and campaigns of local activist groups used to only reach local persons and participants networked in very niche platforms. The strong turn to the digital space of local activists propelled the accessibility and visibility of these events on a worldwide platform. It has also contributed to the worldwide media attention of actions pushed forward and also to establishing transnational connections among other local activist communities.

In fact, recent research on digital activism has been discussing the effects of digitalization in social and political activism, including that of LGBT* activism (Altay, 2022). However, rather than strategically developing and coordinating the digital space and using digital tools to supplement local activism supported by what has been called the transnational advocacy networks (Currier & Moreau, 2016; Keck & Sikkink, 1998), LGBT* organizations in Japan have only recently, if not only since the turn to the digital space in Covid‐19 times, reached out to the digital space. As the exploratory and interdisciplinary literature review of George and Leidner (2019) draws out, digital activism can be found in different forms, yet it is still a nascent field to be further explored. In fact, although the case of Japanese LGBT* activism can be understood in the context of digital activism, the focus of this study rather lies in the contextualization of the activism at the nexus of transnationalization and societal diversification in times of the pandemic and the coinciding mega‐event of the Tokyo Olympics.

## COVID‐19 AND TOKYO OLYMPICS: FROM MOMENT TO MOMENTUM?

One of the main intentions behind hosting mega‐events, despite usually tremendous economic burden, is to bring changes to society, especially economic changes. Investments and subsidies of urban redevelopment projects, infrastructural and public facility developments along with the increase of tourists result in general national and international economic boosts (Malfas et al., 2004; Matheson, 2006). At the same time, issues of social legacy have come into focus of policymakers (Agha et al., 2012). The Tokyo Olympics in 1964 indeed was a cesura in the post‐war Japan history. It helped bring Japan back onto the international stage as a political power and economic rising star, showcasing, inter alia, the technical superiority of its high‐speed Shinkansen trains to the world and also internationalization through introducing street signs and postings in public transportation with transcripts in Latin alphabet. After the ‘Lost Decades’ of Japan's economic recession (Funabashi & Kushner, 2015; Yoshino & Taghizadeh‐Hesary, 2017), it was indeed a clear intention of the government to mark a historical change once again with the hosting of the Olympic Games in 2020. Global attention would bring back investments and corporations to Japan, and the agenda was also to demonstrate the diversity agenda as a global player. As an important side note, the Olympics coincided with new migration policies opening doors to lower skilled migration and proclamation of plans to raise numbers of international students and tourists. While proclaiming the peaceful act of bringing national states together and demonstrating unity, international sporting events have recently come into focus in academic and political debates as they have also been used for political propaganda in history. They have also been scenes of political confrontation—or the suppression of it. Examples reach back to the WW2 and Cold War era, but also recent events, such as the Sochi Olympics or the UEFA soccer games in Hungary illustrate the tension of these international sporting events.

While the motto of ‘Unity in Diversity’ appeared to be a ubiquitous feature of the Olympics and also used for the Tokyo Olympics in 2020, beyond the marketing and the urban redevelopment, not much was planned on the social policy side by the national government (see also critique on such entrepreneurial city marketing and discourses of so‐called homonationalism at sports events, Davidson, 2013; Hubbard & Wilkinson, 2015; Sykes, 2016). However, the LGBT* community in Japan had seized the moment of the Tokyo Olympics and the media attention it was receiving to make it a momentum for a social change with regard to LGBT*‐related diversity issues. Several actions have been taken by LGBT* rights organizations especially during the pandemic, whereas transnational capacities have been used multiply. The novel Covid‐19‐constellation opened up potentials for receiving support beyond local and national borders through digitalization. Furthermore, Covid‐19 caused the postponement of the Tokyo Olympics 2020 by one full year, which has given LGBT* movements even more tailwind as the two following campaigns illustrate. These two cases illustrate how national‐level initiatives headed by originally transnational organizations, such as Human Rights Watch Japan, but also collective initiatives led by local LGBT* groups both used the specific constellation of the pandemic and the mega‐event, and respectively its postponement, for their activism.

### Equality Act Japan

One prime example of an LGBT* rights campaign is the Equality Act Japan, initiated by Human Rights Watch Japan partnering with Japanese local non‐governmental organizations (NGOs) (e.g. Japan Alliance for LGBT* Legislation, Athlete Ally and All Out). The campaign specifically called out for Japan as a host to the Olympic Games to pass an anti‐discrimination law for LGBT* people. It clearly brings the Olympics which ‘represent unity in diversity and passing on a positive legacy for the future’ (Human Rights Watch Japan, 2021) in connection with the lack of a national legislation to protect LGBT* persons, claiming that this ‘fails to meet the requirements of the Olympic Charter, Olympic Agenda 2020’ and generally ‘human rights standards’. As set out on their campaign homepage, the recognition of the momentum for a political change is clear: On the one hand, they point at the ‘Tokyo 2020 games [to be made] a springboard for human rights in Japan and beyond’, but even go further in emphasizing the momentum of the Covid‐19‐caused postponement: ‘The International Olympic Committee and the Japanese government's decision to postpone the games for a year due to the Covid‐19 pandemic gives the entire country the chance to follow Tokyo's lead’ (in passing a bill to prohibit discrimination based on gender identity or sexual orientation in 2018). Furthermore, the interesting take of Human Rights Watch is in their transnational strategy. While the campaign is Japan‐led and primarily focused on mobilizing support for LGBT* rights by the Japanese population, the campaigners strategically aim to also mobilize supporters, that is, petitioners, from abroad to ‘help amplify Japan's call for the quality law’. As clearly referred to by the initiator in an online conversation, creating the so‐called *Gaiatsu* (external pressure) was a conscious choice. This external pressure has been often debated amongst Japanese scholars and foreign policy‐observers as the only mode to make Japanese government relent to major changes in politics (Miyashita, 1999; Pempel, 1999; Tuman & Strand, 2006). Mobilizing domestic and global popular forces, that is, transnationalizing their initiative through online media, was developed as a strategy by this LGBT* campaign through the time won by the Covid‐19‐caused postponement of the Olympics.

### Partnership Act for Tokyo

While the Equality Act Japan is a national‐level initiative that has been strongly transnationalized in times of the Covid‐19 pandemic, local‐level initiatives have also experienced such transnationalization. Though Tokyo had adopted an LGBT* anti‐discrimination bill in 2018, and the aforementioned progress to administratively recognize same‐sex partnerships had begun in wards within the Tokyo prefecture, at the time of the Olympics, there was not yet a prefecture‐wide recognition^3^. The patchwork of local administrations within Tokyo, thus, creates the situation—as a local LGBT* activist put it provocatively—that persons residing in the Western part of the prefecture commuting to the central business district area for work would be administratively changing their partnership status seven times along the way. The grassroot initiative of LGBT* and allies named Partnership Act for Tokyo set up an online petition on Change.org in late January 2021, in the middle of the global pandemic, bringing the situation of hospitalization as an effective and morally obliging case for the recognition of same‐sex partnerships, a clear reference to the dramatic effects of the Covid‐19 pandemic. Translating the petition to English, Chinese and Korean, the initiative posted the call for signing the petition through the transnational network of LGBT* support organizations via social media. They successfully collected more than 18,000 signatures not only from domestic but also global supporters to hand over to the Governor of Tokyo in the spring 2021. With the Tokyo Olympic Games on its way to realization, the pressure had grown for the governor to commit herself to taking steps to introduce the desired prefectural‐wide recognition of same‐sex partnership. As a matter of fact, one of the main initiators of this petition is in fact an LGBT* activist who, with his LGBT* advocacy organization Good Aging Yells, presides over the Pride House Tokyo Consortium. The Pride House Tokyo has been established as part of the Olympic Games’ recent tradition since the 2010 Vancouver Winter Olympic Games to ‘take advantage of the large numbers of people paying attention to the Olympic games’ to promote LGBT* issues and function as a safe space for sexual minorities during but also after the events^4^. Here, too, the connection between the Covid‐19 situation, the mega‐event of Tokyo Olympics and transnationalization, especially through global digital connectedness, is evident.

**FIGURE 1:**
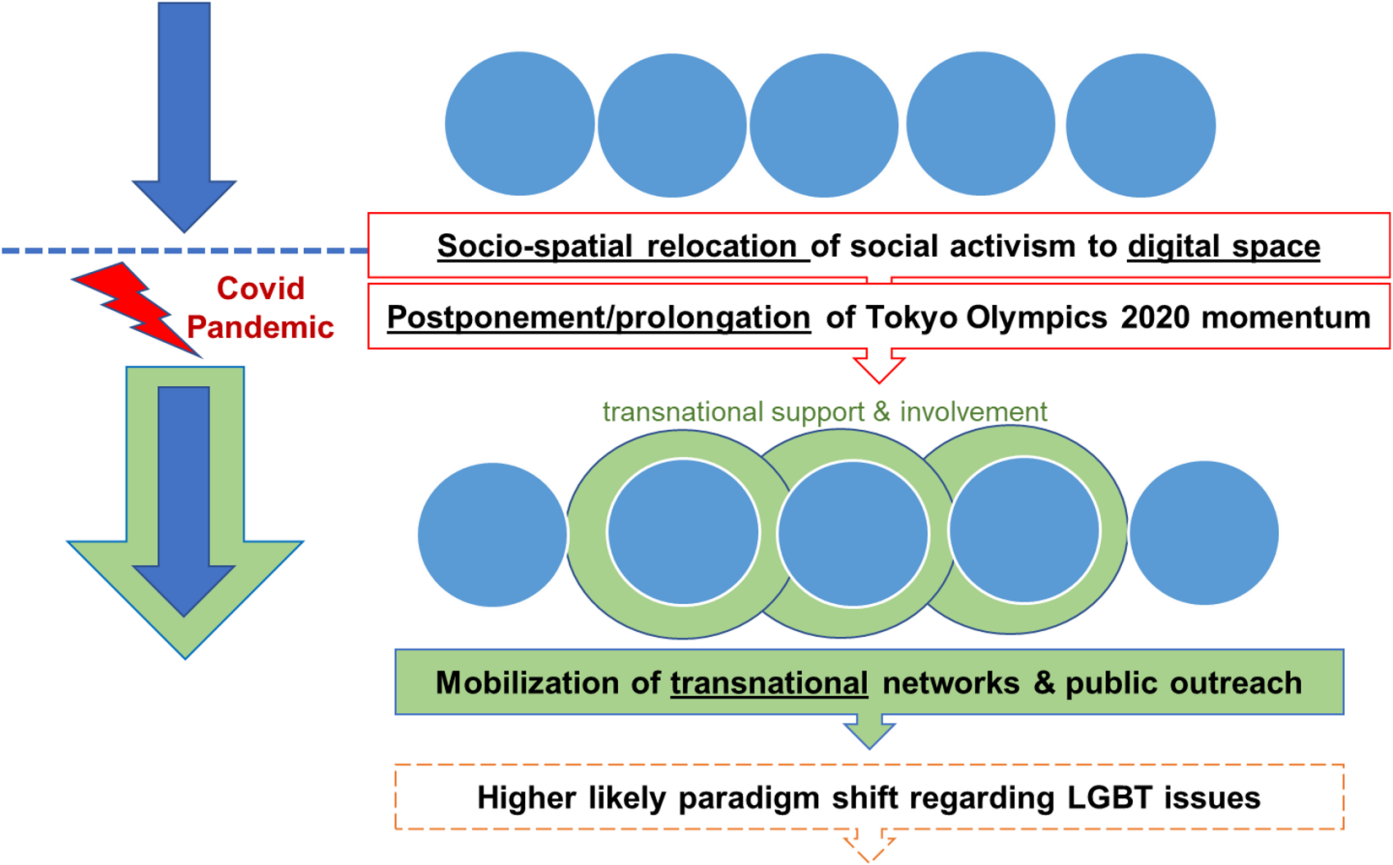
Impact of the Covid‐19 pandemic on the transnationalization of local LGBT* activism in Japan

Summing up these movements from a conceptual perspective, two main changes were introduced through Covid‐19: the socio‐spatial relocation of LGBT* activism to the digital space, and the postponement of the Tokyo Olympics 2020 which has prolonged and enabled activists to mobilize people domestically and globally (Figure 1). The observations show that Covid‐19 may not be the actual cause and driver of these social transformations. However, it can be said with certainty that the digitalization and spatial relocation of local social practices and activism into the digital world caused by the pandemic led to the acceleration and enhancement of transnationalization of actions taken by LGBT* organizations. By the involvement of actors beyond national borders, they continue to reach greater audiences and attention from the media worldwide. Indeed, the current momentum of the LGBT* movement in Japan is essentially contextualized in the Covid‐19 pandemic.

## COVID‐19 ENHANCED TRANSNATIONALIZATION IN LGBT* COMMUNITIES

Although the impact of such transnational support for local movements tends to be difficult to grasp and quantify, the case of the local court in the legal cases on same‐sex marriages shows how impactful they can be. In the landmark decision of the Sapporo district court on 17 March 2021, declaring that the nonrecognition of same‐sex marriage is unconstitutional, two interesting references were made for the judgment.

One was the reference to the increasing support of the local population for same‐sex marriages, reflected in the polls of surveys conducted by larger new media outlets, giving grounds to protect minority issues in the majority society. Observing Tokyo Rainbow Pride, the transnational experiences and practices of Japanese but also mixed‐ethnicity Japanese public figures, private persons and corporate actors, appear to be clearly contributing to such visibility of diversity and also acceptance of this diversity. As examples of digital ethnography that supports this argument on transnationalization and growing support of LGBT* issues among the general public, interviews during the Tokyo Rainbow Pride were illustrative. Both, a former announcer and employee within one of the largest private TV stations and the Executive Director of the main sponsoring corporation, stated how much their own overseas experiences as correspondents from the overseas news bureau, as student of business studies on Diversity & Inclusion (D&I) in Northern America, have impacted their understanding of diversity issues. Similarly, the narrative of binational and bilingual YouTubers and other public figures who are supportive of the pride movement stressed what can be regarded as an important aspect of transnationalization in society. They reflected on the linguistic dimensions of LGBT* terminologies, which had been observed academically as the ‘LGBT* boom’ or the paradigm shift to the Anglicization of queer issues. They also discussed their realization of previous ignorance on diversity issues when moving or traveling abroad, showcasing how the awareness for diversity has grown in the transnational context. Based on their transnational life experiences and lived realities, they now act as spokespersons for gender and sexual diversities, contributing to the growing acceptance of LGBT* diversity in society

The other reference that was made to assess the importance of accepting same‐sex marriages from the legal point‐of‐view were the recommendations of five foreign Chambers of Commerce on the economic viability of diversity in society and their urge to legalize same‐sex marriage in Japan. Realizing the potential of such economic perspectives on social transformation, local and transnational LGBT* organizations have started campaigns to win more companies to back the legalization of same‐sex marriage, signalling the politics to change the legislation. The Business for Marriage Equality, a collaborative campaign of the non‐profit organizations Marriage for All Japan (MFAJ), Nijiiro Diversity and Lawyers for LGBT* & Allies Network (LLAN), endorses the acceptance of diversity and implementation of diversity agenda in firms. As activists point out, most of the 160 transnational corporations are more dominantly represented in these lists, but with multinational Japanese companies opting in, they aim to persuade also more local firms to sign up.

Additionally, it is also striking how transnational these NGOs and NPOs themselves already are. Activists and professionals involved in them show a mix of different nationalities, but they also actively build bridges between local and transnational LGBT* communities. As it can be seen in transnational LGBT* business networks, such as Fruits in Suits, or the Pride Business Alliance, Japan's first LGBTQIA+ Chamber of Commerce, they are led by non‐Japanese actors. In fact, even the Japanese lawyers involved in the court cases are Japan and US (New York bar) accredited, thus being transnational professionals, and their plaintiffs partly also being binational couples. It appears that transnationalization is already a lived reality in the LGBT* community. Due to these transnational constellations and contexts at different levels, many of the above‐mentioned digital events were offered bilingually, in Japanese and English. This transnationalization appears to be contributing indeed to the superdiversification of societies, and now through the Covid‐19 pandemic, has won further drive for public support locally and globally.

In spite of these optimistic trends to a social transformation involving more diversity, the activists’ high hopes have recently been damped once again by the leading LDP politicians. LDP politicians questioning the LGBT*’s existence and not passing the anticipated anti‐LGBT* discrimination law that was supposed to be implemented with the Tokyo Olympic Games seem to have halted the momentum of LGBT* actions before the mega‐event. Indeed, with the dwindling support for the Tokyo Olympic Games in general, especially in view of the national government's lack in getting hold of the Covid‐19 pandemic, it had become questionable if the much‐desired impulse of the mega‐event to mark the turning point for LGBT* rights in Japan could be realized. However, the trend of the social transformation to greater diversity is in striking distance, and very likely to be reached with the appeals to the Supreme Court in couple of years. Though the outcome of the debates is still open, there is a clear connection between the transnationalization of local social and political activism catalysed by Covid‐19‐induced digitalization and transnationalization. Without the Covid‐19 pandemic, it is highly likely that the media attention and public outreach for LGBT* activism, which have been achieved by the transnational connections built through the digital, would not have been as large, or won such a momentum at all in the first place.

## IMPACT OF COVID‐19 ON THE FUTURE OF A GLOBAL COMMUNITY

The Covid‐19 pandemic has been an important catalyst for transnationalization of the local LGBT* movement in Japan, evidently pushing its spatial boundaries to achieve broader public outreach, but in turn, also stronger support from beyond the national border. The LGBT* movement is however, only one such example and the increased transnationalization of events, involving not only local but also global actors, can also be observed in further diversity‐related organizations and even in the start‐up scene in Japan. The Covid‐19 pandemic appears to have accelerated the ubiquity, and thus availability and accessibility of digital events, leading to an even more connected and sustainable social transformation to diversity. Yet, such change needs to be contextualized with the coinciding global media attention and the external pressure which occurred through the (postponed) Olympic Games. In such a context, the question of the general external pressure ‘gaiatsu’ created through the transnationalization and digitalization of LGBT* activism could be further revisited. The interesting point, however, to consider is the already lived reality of diversity rather than the political instrumentalization otherwise connected to the gaiatsu. Contemporary society with its transnational couples, lawyers, activists, but also corporations, clearly creates a transnational, that is, in its existence bridging two or more national contexts, type of pressure. As these transnational pressures are neither simply internal nor external, but actually inherently also local, its potential for pushing societal changes could be increasing in the near future. The pandemic was not the driver of these novel development in society and neither was the Olympics; it was consciously used as a crucial moment and turned into a momentum. These occasions, which serve as the analytical focus here, crystallize the already ongoing transnationalization of LGBT* movements, reflecting the general migration‐led diversification of societies.

## CONCLUSION

Returning to the critical reflection on the discourses of global networks and transnationalism, there are several implications and possible avenues for future studies.

One apparent issue is the already‐hinted change of the spatiality in social interactions and encounters, or the spatiality of superdiversity (Yamamura, 2022). Beyond debates on changing boundaries or the merging of online and offline spaces and networks (e.g. Blommaert, 2015), or specifically LGBT* urban spaces (Bain & Podmore, 2021), there is a need to further look into the general transnational social practices that have been enabled or at least accelerated through Covid‐19‐induced digitalization (cf. also the call for the geographical perspective in Nagel & Staehli, 2010). Such digitalization has been indeed discussed by the emerging field of digital geography (Ash et al., 2018; Nagel & Staeheli, 2010) and by scholars on transnational migrations, such as Van den Bos and Nell (2006) in the context of de‐/territorialization of transnational migrants through new media. Yet, the novel phenomenon which needs to be more looked into in this context of spatiality of social interactions and encounters is to methodologically properly break with ethnic and national focality (Meissner & Vertovec, 2015; Yamamura & Lassalle, 2020), which is still found widely in transnationalism research. Transnationalism research has identified already decades ago that there is a new social field establishing through transnational migration, with communities bridging two or even more locations. Yet, research still tends to focus on the multilocality of specific ethnic or national groups rather analysing the communities themselves that are transnationalized and go beyond the bi‐ or multilocality of one group, and neglect the actual transnational nature of communities. As can be seen in the novel development in the LGBT* activism, the local community does not only rely on Japanese networks abroad, but on strongly diverse and truly transnational groups beyond the ethnic and national groupism framing. The transnational character of the LGBT* community, and with that, transnational spaces, lies in the actual lived realities of transnationally living bi‐national couples, transnational LGBT* persons and allies, as well as a global community of people from different countries united by the LGBT* attribute rather than their ethnic or national identities. Such commonality of identification of transnational communities can be also observed in business networks and transnational corporate migrants (Yamamura, 2022). Such examples call for revisiting the actual transnational dimension in the novel context of the increase in migration‐led diversification of societies around the globe and to further delve into the novel socio‐spatial dynamics that increasing superdiversity is bringing to society and space.

Moreover, the question of the digital divide for global connectedness is another issue to further explore. As scholars have discussed before, the ubiquity of virtual events does not necessarily correlate with actual accessibility. On the one hand, the digital divide with regard to access to internet communication media and digital devices remains a prime cause for inequality (Dodge & Kitchin, 2003; Graham et al., 2014; Norris, 2020). Yet, the transformation of in‐presence events to digital events has made resources more available for much lower costs than before. On the other hand, what needs to be considered more is the temporality of interactions which has been accelerated by Covid‐19‐induced digitalization and transnationalization. In fact, the time difference in the organization of such events is bringing another novel take on the digital divide. As LGBT* movements but also further diversity‐related events have shown, the collaborations of LGBT* activists in the form of live events are strongly limited to the time differences that can be accommodated. While the novel digital platform has contributed to a stronger interaction and exchange between LGBT* communities worldwide, there is a novel East‐West time‐divide occurring. While German local groups have begun bridging their spatial boundaries of being isolated from larger metropolises and co‐hosting with novel partners from Eastern Europe or with East African activists, the time difference limits the possibility to collaborate with, for example, Southeast‐Asian or Norther American activists from the Pacific rim. The transnationalization of activism propelled by Covid‐19 appears to bridge the North‐South divide and bears important potential of collaborating transnationally, contributing to a far too neglected global connection to the Global South. Yet, there are temporal aspects that are creating novel spatio‐temporal borders on the East‐West axis.

The changes in everyday life caused by the Covid‐19 pandemic appear to go beyond simple digitalization and are strongly interwoven with the global connectedness and transnationalization of societies. Covid‐19 has created new potential for social and political activism to contribute to social transformations by drawing on the resources of such transnational networks. While such interwoven spatial and temporal dynamics need to receive further attention in future research on transnationalism and global connectedness, revisiting transnationalism from a superdiversity perspective appears to be essential. This critical reflection on recent developments in Japanese LGBT* activism in times of the pandemic has illustrated only one such societal diversification which has become inherently transnational.

## CONFLICT OF INTEREST

The author declares no conflict of interest.

## Data Availability

Data available on request from the authors.

## Footnotes

1 At the time of the initial submission of the manuscript, the Olympic Games were still in planning. While novel changes in policies and administrative procedures have been incorporated, the main reference of the empirical study focuses on the time up until the hosting of the Olympics in the summer 2021.

2 The capital of the northern‐most prefecture Hokkaido.

3 At the time of the final submission of this manuscript, in fact, the Governor of Tokyo Koike who had successfully maintained her position at the general election in October 2021 has proclaimed her commitment to propose the recognition of same‐sex partnership to the city council within the new fiscal year starting in April 2022. Starting on 1 November 2022, Tokyo prefecture issues same‐sex partnership certificates.

4 As a side note, such Pride Houses have been established during each of the Olympic Games ever since and have become even more present following the Russian government's anti‐gay propaganda law prior to the 2014 Sochi Winter Olympics which caused an uproar worldwide. Sexual orientation was added to the anti‐discrimination clause of the IOC Charter as a consequence in 2015. Managed primarily by local NGOs and NPOs, these Pride Houses are organized as a transnational network named Pride House International.
